# Astrocyte-gated multi-timescale plasticity for online continual learning in deep spiking neural networks

**DOI:** 10.3389/fnins.2025.1768235

**Published:** 2026-01-27

**Authors:** Zhengshan Dong, Wude He

**Affiliations:** 1 School of Computer and Data Science, Minjiang University, Fuzhou, China; 2 Metatop (Fuzhou) Technology Co., Ltd., Fuzhou, China

**Keywords:** astrocytes, continual learning, eligibility traces, neuromorphic computing, online learning, spiking neural networks, synaptic plasticity, three-factor learning

## Abstract

Spiking Neural Networks (SNNs) offer a paradigm of energy-efficient, event-driven computation that is well-suited for processing asynchronous sensory streams. However, training deep SNNs robustly in an online and continual manner remains a formidable challenge. Standard Backpropagation-through-Time (BPTT) suffers from a prohibitive memory bottleneck due to the storage of temporal histories, while local plasticity rules often fail to balance the trade-off between rapid acquisition of new information and the retention of old knowledge (the stability-plasticity dilemma). Motivated by the tripartite synapse in biological systems, where astrocytes regulate synaptic efficacy over slow timescales, we propose Astrocyte-Gated Multi-Timescale Plasticity (AGMP). AGMP is a scalable, online learning framework that augments eligibility traces with a broadcast teaching signal and a novel astrocyte-mediated gating mechanism. This slow astrocytic variable integrates neuronal activity to dynamically modulate plasticity, suppressing updates in stable regimes while enabling adaptation during distribution shifts. We evaluate AGMP on a comprehensive suite of neuromorphic benchmarks, including N-Caltech101, DVS128 Gesture, and Spiking Heidelberg Digits (SHD). Experimental results demonstrate that AGMP achieves accuracy competitive with offline BPTT while maintaining constant O(1) temporal memory complexity. Furthermore, in rigorous Class-Incremental Continual Learning scenarios (e.g., Split CIFAR-100), AGMP significantly mitigates catastrophic forgetting without requiring replay buffers, outperforming state-of-the-art online learning rules. This work provides a biologically grounded, hardware-friendly path toward autonomous learning agents capable of lifelong adaptation.

## 1 Introduction

Spiking Neural Networks (SNNs) have emerged as a promising paradigm for energy-efficient machine intelligence, offering a computational substrate that closely mimics the sparse, event-driven dynamics of biological brains (Maass, 1997; Roy et al., 2019). Unlike traditional Artificial Neural Networks (ANNs) that process continuous-valued activations synchronously, SNNs communicate via binary spikes asynchronously. This temporal sparsity is particularly advantageous when processing dynamic sensory streams from neuromorphic hardware, such as Dynamic Vision Sensors (DVS) and silicon cochleas, where information is encoded in the precise timing of events rather than static frames (Gallego et al., 2022). As the demand for edge computing and low-latency deployment grows, developing deep SNNs that can learn robustly and efficiently on neuromorphic chips has become a central objective in the field.

However, training deep SNNs to high accuracy remains a formidable challenge due to the non-differentiable nature of the spike generation function. The current state-of-the-art approach, Backpropagation-through-Time (BPTT) with surrogate gradients, circumvents this issue by smoothing the derivative of the Heaviside step function (Wu et al., 2018; Neftci et al., 2019). While BPTT has enabled SNNs to achieve performance competitive with ANNs on complex recognition tasks, it is fundamentally an offline and memory-intensive algorithm. BPTT requires unrolling the network over the entire simulation time window *T* and storing the intermediate membrane potentials of all neurons to compute gradients. This results in a memory complexity that scales linearly with time (O(T)), creating a prohibitive “memory wall” for processing long temporal sequences or for deploying learning algorithms directly on resource-constrained edge devices (Zenke et al., 2021). Furthermore, BPTT assumes that the entire dataset is available for repeated offline epochs, a condition that holds for static benchmarks but fails in real-world scenarios where data arrives in a continuous, non-stationary stream.

To address the limitations of offline BPTT, significant research effort has shifted toward synapse-local plasticity rules inspired by biological mechanisms, such as Spike-Timing-Dependent Plasticity (STDP) (Dan and Poo, 2004). While classical pair-based STDP is computationally efficient and locally implementable, it often struggles to solve complex credit assignment problems in deep, hierarchical networks. Recent advances in “three-factor” learning rules, which combine local presynaptic and postsynaptic activities with a global modulatory signal (e.g., error or reward), have begun to bridge the gap between biological plausibility and deep learning performance (Frémaux and Gerstner, 2016). Notably, the eligibility propagation (e-prop) framework (Bellec et al., 2020) provides a mathematical justification for online learning, factoring the gradient into a fast, synapse-local eligibility trace and a broadcast learning signal. These methods allow weights to be updated at every time step without storing the full history of neural states, theoretically enabling online learning with constant memory complexity (O(1)).

Despite these advances in online learning, a critical unresolved issue is Continual Learning (CL). In realistic deployment scenarios, an intelligent agent must learn a sequence of tasks or adapt to changing data distributions over time. When standard plasticity rules (including three-factor methods) are applied to sequential tasks, they suffer from “catastrophic forgetting, where the adaptation to new information rapidly overwrites previously acquired knowledge” (Kirkpatrick et al., 2017; Parisi et al., 2019). In the ANN literature, this stability-plasticity dilemma is often addressed using replay buffers or regularization techniques that require computing the Fisher Information Matrix (e.g., Elastic Weight Consolidation). However, these methods are computationally heavy and often incompatible with the strict locality and online constraints of neuromorphic hardware. Achieving robust continual learning in SNNs requires a mechanism that can regulate plasticity dynamically, stabilizing important synapses while allowing others to adapt, without relying on external memory buffers or offline batch processing.

Theoretical neuroscience suggests that the stability of biological memory arises from the interaction of multiple processes operating across distinct timescales (Benna and Fusi, 2016; Zhang et al., 2022). While neurons and synapses operate on the millisecond scale, non-neuronal glial cells, particularly astrocytes, regulate synaptic function on timescales of seconds to minutes. The concept of the “Tripartite Synapse” posits that astrocytes are active partners in computation, integrating neuronal activity and releasing gliotransmitters that gate synaptic plasticity (Perea et al., 2009; Stogsdill and Eroglu, 2017). This slow, homeostatic regulation serves as a natural mechanism for metaplasticity, determining when and how much a synapse should change based on the broader context of neuronal activity.

Motivated by these biological principles, we propose Astrocyte-Gated Multi-Timescale Plasticity (AGMP), a novel online learning framework for deep SNNs. AGMP extends the three-factor eligibility trace paradigm by introducing a fourth factor: an astrocyte-like slow state variable that integrates local neuronal activity. This slow variable acts as a dynamic gate for synaptic updates, effectively modulating the learning rate based on the historical activity context of the neuron. By coupling fast eligibility traces (for temporal credit assignment) with slow astrocytic gating (for stability) and a broadcast error signal (for task performance), AGMP enables deep SNNs to learn continuously from streaming data. We demonstrate that this multi-timescale approach not only matches the accuracy of offline BPTT on event-based neuromorphic benchmarks, such as DVS128 Gesture and SHD, but also significantly mitigates catastrophic forgetting in Class-Incremental and Task-Incremental learning scenarios. This work provides a scalable, biologically interpretable path toward robust online learning in neuromorphic systems, ensuring more trustworthy computing in decision-making (Song and Zhang, 2025).

This paper makes the following contributions:

- *A structured four-factor learning mechanism*. We introduce an astrocyte-like slow state that gates eligibility-trace learning driven by a broadcast modulatory teaching signal, yielding an online four-factor plasticity rule: eligibility × modulation × astrocytic gate × stabilization.
- *Deep and online learning without BPTT histories*. AGMP maintains only synapse-local eligibility traces and neuron-local astrocyte states, avoiding the need to store *T*-step histories typical of BPTT and enabling scalable online updates in deep CNN-SNN and spiking ResNet architectures.
- *Continual learning evaluation under Task-IL and Class-IL*. We evaluate AGMP under both task-incremental (multi-head) and class-incremental (single-head) protocols (van de Ven and Tolias, 2019), highlighting the role of astrocytic gating in reducing interference under the more challenging Class-IL setting.
- *Energy-aware reporting and mechanistic ablations*. In addition to accuracy, we report spike counts and synaptic operations (SynOps) as energy proxies, and we include ablations (gate, eligibility, homeostasis) and time-scale sensitivity sweeps to isolate the contributions of each component.

## 2 Related work

### 2.1 Deep SNN training with surrogate gradients

Training SNNs has historically been impeded by the non-differentiable nature of discrete spike events. Early approaches largely relied on ANN-to-SNN conversion (Diehl et al., 2015; Sengupta et al., 2019; Zhang et al., 2024), where a rate-coded SNN approximates a pre-trained ANN. While conversion methods yield high accuracy on static image datasets, they typically suffer from high inference latency and fail to capture the rich temporal dynamics required for event-based sensory processing.

To enable direct training in the temporal domain, the concept of Surrogate Gradients (SG) was introduced (Neftci et al., 2019; Zenke and Ganguli, 2018; Wu et al., 2018). By replacing the derivative of the Heaviside step function with a smooth auxiliary function (e.g., sigmoid or arctangent) during the backward pass, SG allows SNNs to be optimized via Backpropagation-through-Time (BPTT). This paradigm has established the current state-of-the-art for deep SNNs on complex neuromorphic benchmarks (Shrestha and Orchard, 2018; Yin et al., 2020; Wu et al., 2025). However, BPTT is inherently non-local in time; it requires unfolding the network graph and storing synaptic and membrane states for the entire duration of the input sequence. This results in a memory complexity of O(T·N), rendering the training of long sequences on memory-constrained edge devices computationally prohibitive (Zenke et al., 2021).

### 2.2 Online learning and eligibility traces

To overcome the “memory wall” of BPTT, recent research has pivoted toward forward-mode learning rules that rely on local approximations of the gradient. Inspired by the biological three-factor learning rule, which involves presynaptic activity, postsynaptic state, and a global modulator (Frémaux and Gerstner, 2016; Porr and Wörgötter, 2007), researchers have formalized these dynamics into efficient algorithms.

The e-prop algorithm (Bellec et al., 2020) represents a significant breakthrough, mathematically decomposing the gradient into a synapse-local eligibility trace and a broadcast learning signal. This allows weights to be updated online at every time step without storing future gradients. Similar approaches, such as Online Spatio-Temporal Learning (OSTL) (Bohnstingl et al., 2023) and Super-Spike (Zenke and Ganguli, 2018), share this philosophy of separating temporal credit assignment (trace) from spatial error assignment. While these methods achieve O(1) temporal memory complexity, they primarily address the online aspect of learning rather than the continual aspect. When exposed to non-stationary data streams without explicit regularization, these plasticity rules are prone to rapid weight overwriting, leading to performance degradation on previously learned tasks.

### 2.3 Continual learning and stability-plasticity

Continual Learning (CL) deals with learning from a stream of data where the distribution changes over time, posing the “stability-plasticity dilemma” (Parisi et al., 2019). In the context of ANNs, prominent strategies include regularization-based methods like Elastic Weight Consolidation (EWC) (Kirkpatrick et al., 2017) and Synaptic Intelligence (SI) (Zenke et al., 2017), which penalize changes to parameters critical for past tasks. However, these methods typically require calculating the Fisher Information Matrix or path integrals, which are computationally expensive to estimate in a purely online, streaming setting.

In SNNs, CL is often approached via architecture expansion (Rusu et al., 2022) or replay buffers (Rebuffi et al., 2017), but these solutions entail growing memory costs that negate the efficiency benefits of neuromorphic hardware. Recent efforts have explored metaplasticity—the plasticity of plasticity itself. For instance, models incorporating hidden synaptic states (Benna and Fusi, 2016) or binary synapses with probabilistic transitions (Fusi et al., 2005) can extend memory retention. Nevertheless, integrating these theoretical mechanisms into deep, supervised SNNs capable of processing high-dimensional inputs (e.g., DVS gestures) remains an open challenge. Our work addresses this by implementing metaplasticity not through hidden weights, but through an explicit, biologically grounded gating variable.

### 2.4 Astrocytes in neuromorphic computing

Biological synapses are not merely bipartite connections between neurons; they are tripartite structures sheathed by astrocytes (Araque et al., 1999; Perea et al., 2009). Neuroscience evidence suggests that astrocytes integrate neuronal activity over slow timescales (seconds) and release gliotransmitters (e.g., glutamate, D-serine) that regulate the threshold for synaptic plasticity (Bernardinelli et al., 2014; Stogsdill and Eroglu, 2017).

Computational models of neuron-glia interactions have traditionally focused on unsupervised tasks, such as self-repair (Wade et al., 2012), synchronization (Chen and Li, 2024), or unsupervised feature clustering (Shen et al., 2023). More recently, researchers have begun to explore astrocytes for supervised learning. For example, Rastogi et al. (2021) proposed astrocyte-modulated STDP to stabilize learning rates, and Reichenbach et al. Reichenbach et al. (2010) demonstrated that glial-like regulation can extend memory retention in simple networks. However, these works often rely on shallow architectures or simplified tasks. AGMP distinguishes itself by mathematically formulating the astrocyte as a gating factor for SG within an eligibility trace framework, enabling scalable, supervised continual learning in deep convolutional and recurrent SNNs.

## 3 Proposed method

In this section, we present the theoretical formulation of Astrocyte-Gated Multi-Timescale Plasticity (AGMP). We begin by defining the underlying spiking neuron models and the problem of gradient-based learning in discrete time. We then derive the online learning rule using eligibility traces and introduce the novel astrocytic gating mechanism designed to regulate plasticity dynamics for continual learning stability.

### 3.1 Spiking neuron dynamics

We adopt the Leaky Integrate-and-Fire (LIF) model and its adaptive variant (ALIF) as the computational units. These models capture the essential temporal dynamics of biological neurons while remaining computationally tractable for deep network simulation.

Consider a deep SNN with layers ℓ = 1, …, *L*. The membrane potential [t]iℓ of neuron *i* in layer ℓ at discrete time step *t* evolves according to:


[t+1]iℓ=αiℓ[t]+∑jwijjℓ ℓ−1[t]+biℓ−ʒiℓ[t]iℓ[t], (1)

where α = exp(−Δ*t*/τ_*m*_) represents the leakage factor with membrane time constant τ_*m*_, $w_{ij}^{\ell }$ is the synaptic weight from presynaptic neuron *j*, $b_{i}^{\ell }$ is a bias term, and [t]jℓ−1∈{0,1} denotes the input spike. The output spike [t]iℓ is generated via a deterministic threshold mechanism:


[t]iℓ=H(iℓ[t]−ʒiℓ[t]), (2)

where *H*(·) is the Heaviside step function. Following a spike, the membrane potential is reset by subtracting the threshold value (soft reset). To enhance temporal processing capabilities, particularly for datasets like SHD, we employ Adaptive LIF (ALIF) neurons. In ALIF, the threshold ${ʒ}_{i}^{\ell }\left[t\right]$ is dynamic:


$$\begin{array}{l} {ʒ}_{i}^{\ell }\left[t+1\right]={ʒ}_{0}+\rho \left({ʒ}_{i}^{\ell }\left[t\right]-{ʒ}_{0}\right)+\beta_{i}^{\ell }\left[t\right], \end{array}$$ (3)

where ʒ_0_ is the baseline threshold, ρ determines the decay of the adaptation, and β controls the magnitude of threshold increase post-spike. This adaptation effectively models firing rate adaptation, providing the network with a longer working memory.

### 3.2 Gradient descent with eligibility traces

The core challenge in training SNNs is the non-differentiable nature of the discrete spike function *S*(·). We utilize the surrogate gradient method (Neftci et al., 2019), where the derivative is approximated during learning by a smooth function ψ(·), such as a piecewise linear or sigmoid function:


∂iℓ[t]∂iℓ[t]≈ψ(iℓ[t])=γmax(0,1−|[t]iℓ−ʒδ|). (4)

Standard BPTT computes the exact gradient of the loss L by unrolling the network over time. However, this violates the requirement for online learning. To achieve locality in time, we adopt the factorization proposed in the *e-prop* framework (Bellec et al., 2020). The gradient of the loss with respect to a weight *w*_*ij*_ at time *t* is approximated as the product of a global learning signal and a local eligibility trace:


$$\begin{array}{l} \frac{\partial L}{\partial w_{ij}^{\ell }}\approx \underset{t}{\sum }\underset{\text{Learning Signal}}{\underset{︸}{L_{i}^{\ell }\left[t\right]}}\times \underset{\text{Eligibility Trace}}{\underset{︸}{e_{ij}^{\ell }\left[t\right]}}. \end{array}$$ (5)

The eligibility trace $e_{ij}^{\ell }\left[t\right]$ maintains a fading memory of the correlation between presynaptic input and postsynaptic state. For LIF neurons, this is derived recursively:


$$\begin{array}{l} e_{ij}^{\ell }[t+1]=\lambda_{e}e_{ij}^{\ell }[t]+\psi {{(}_{i}^{\ell }[t])}_{j}^{\ell -1}[t], \end{array}$$ (6)

where λ_*e*_ relates to the membrane time constant. This variable is purely local to the synapse (*i, j*) and can be computed forward in time. For the learning signal, we employ a top-down error broadcast. In the output layer, $M_{i}^{L}\left[t\right]=y_{i}\left[t\right]-{ŷ}_{i}\left[t\right]$. For hidden layers, exact backpropagation of error requires symmetric weight transport (*W*^*T*^), which is biologically implausible. We instead use direct error broadcast, projecting the global error vector ***δ***[*t*] to hidden neurons via fixed random matrices *B*^ℓ^:


$$\begin{array}{l} M_{i}^{\ell }\left[t\right]=\underset{k}{\sum }B_{ik}^{\ell }\delta_{k}\left[t\right]. \end{array}$$ (7)

This signal $M_{i}^{\ell }\left[t\right]$ serves as the “third factor” in the plasticity rule, informing the neuron of its contribution to the global objective. To ensure the error signal remains within a range that the astrocyte-gated update can effectively regulate, the elements of the fixed random matrices *B*^ℓ^ are initialized using a variance-preserving heuristic (scaled by $1/\sqrt{N_{in}}$), preventing the gate from struggling to converge due to initially exploding error magnitudes.

### 3.3 Astrocyte-gated multi-timescale plasticity (AGMP)

**Figure 1:**
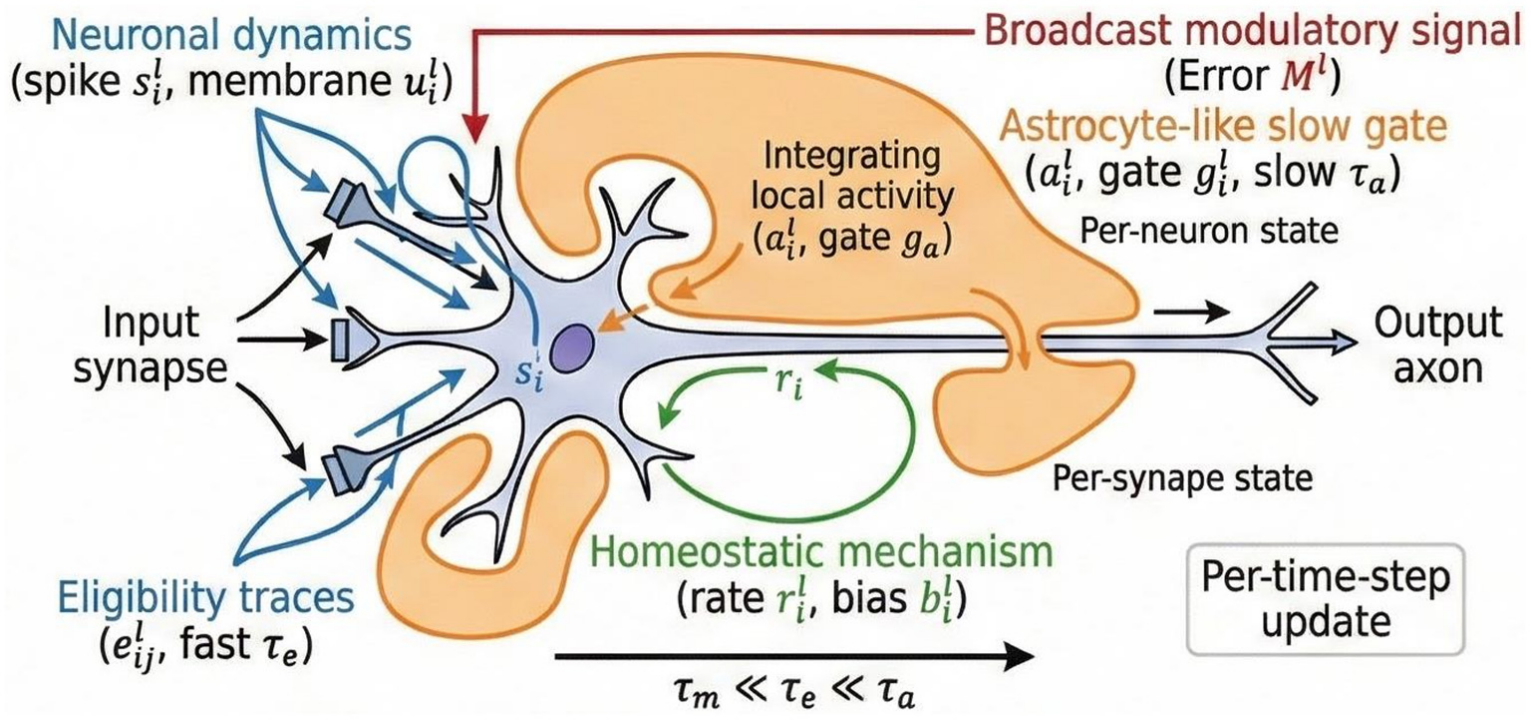
Schematic of Astrocyte-Gated Multi-Timescale Plasticity (AGMP). The framework mimics the tripartite synapse. Neuronal dynamics (blue) operate on a fast timescale (τ_*m*_). Synaptic eligibility traces (light blue) capture millisecond correlations (τ_*e*_). The astrocyte (orange) integrates local activity over a slow timescale (τ_*a*_) to compute a gating factor *g*_*i*_. This gate modulates the update driven by the broadcast error signal (red), enabling the network to balance plasticity (during new tasks) and stability (during consolidation).

While the three-factor rule described above enables online learning, it treats all updates with equal plasticity potential, leading to catastrophic forgetting in non-stationary environments. We propose AGMP as shown in Figure 1, which introduces a fourth factor: a slow, astrocyte-mediated gating variable.

#### 3.3.1 Biological motivation and dynamics

In the brain, astrocytes ensheath synapses and regulate their efficacy. Unlike neurons, which operate on the millisecond timescale, astrocytes integrate activity over seconds to minutes. We hypothesize that this slow integration provides a robust estimate of the contextual stability or metabolic cost of a circuit, which can be used to modulate plasticity rates dynamically. We associate a scalar astrocyte state $a_{i}^{\ell }\left[t\right]$ with each neuron *i*, which evolves on a slow timescale τ_*a*_ ≫ τ_*m*_, τ_*e*_:


$$\begin{array}{l} a_{i}^{\ell }\left[t+1\right]=\lambda_{a}a_{i}^{\ell }\left[t\right]+\left(1-\lambda_{a}\right)\phi_{i}^{\ell }\left[t\right], \end{array}$$ (8)

where λ_*a*_ = exp(−Δ*t*/τ_*a*_). The drive function $\phi_{i}^{\ell }\left[t\right]$ represents the local activity load:


ϕiℓ[t]=ηu|iℓ[t]|+ηs∑j|wijℓ|jℓ−1[t]+ηo[t]iℓ. (9)

where η_*u*_, η_*s*_, and η_*o*_ are positive scaling coefficients that control the relative contribution of membrane potential, synaptic input, and output spikes, respectively. This formulation implies that the astrocyte integrates presynaptic drive, postsynaptic potential, and output spiking. A high value of *a*_*i*_ indicates a neuron that has been consistently active or highly stimulated over the recent past.

#### 3.3.2 Context-aware gating and update rule

**Figure 2:**
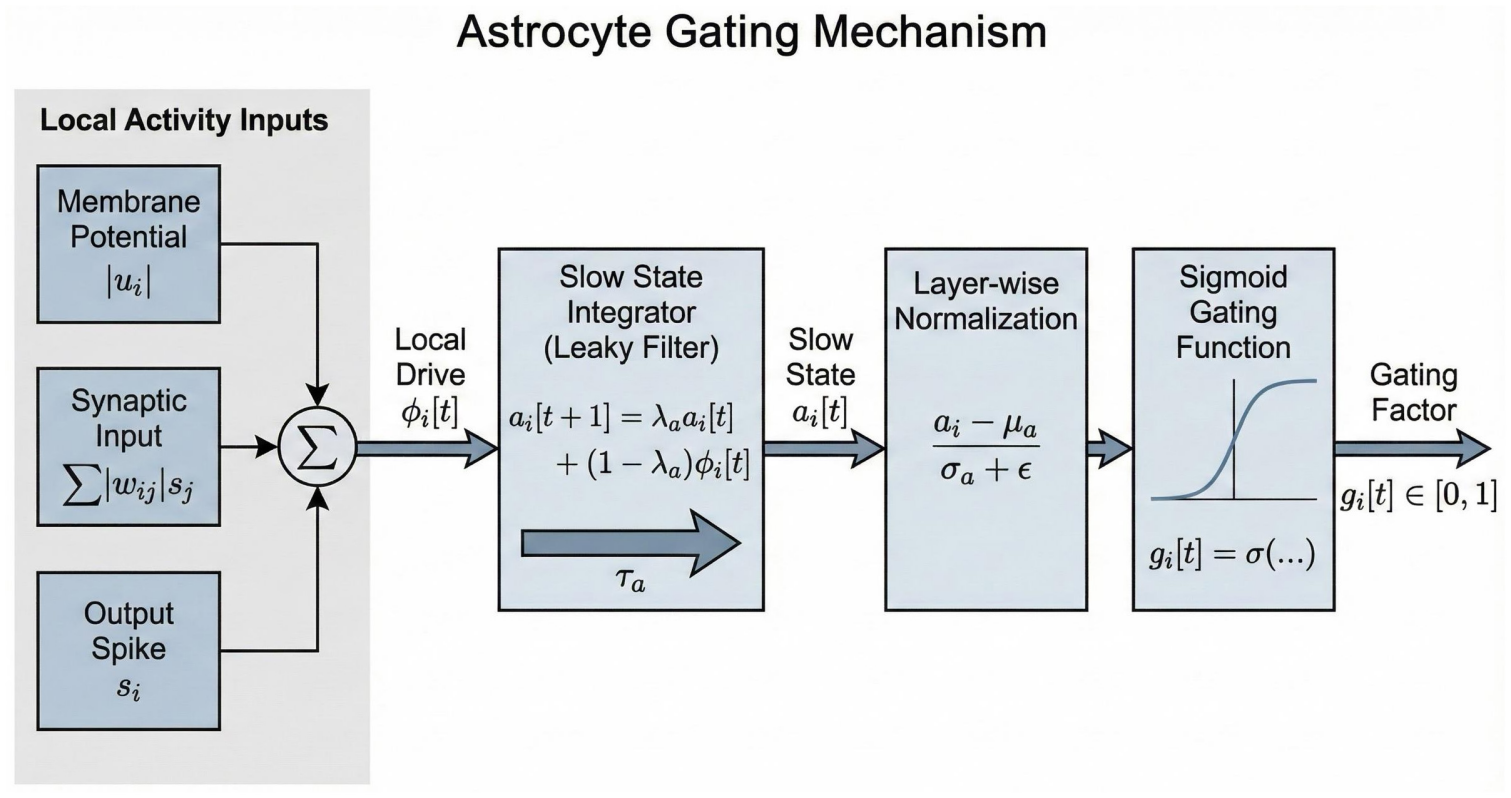
Astrocyte Gating Mechanism. The local activity inputs (membrane potential, synaptic input, and output spikes) drive a slow state integrator. This slow state *a*_*i*_ is normalized and passed through a sigmoid function to produce the gating factor *g*_*i*_∈[0, 1]. This gate essentially determines the ”write permission” for the synaptic weights connected to neuron *i*.

To convert the astrocyte state into a plasticity modulator, we employ a normalization and gating function (Figure 2). At *t* = 0, the astrocyte state is initialized to $a_{i}^{\ell }\left[0\right]=0$. We then compute the layer-wise moving statistics (mean $\mu_{a}^{\ell }$ and variance ${\sigma_{a}^{\ell }}^{2}$) of the astrocyte states. The normalized state is ${â}_{i}^{\ell }\left[t\right]=\left(a_{i}^{\ell }\left[t\right]-\mu_{a}^{\ell }\right)/\left(\sigma_{a}^{\ell }+\epsilon \right)$. The gating factor $g_{i}^{\ell }\left[t\right]$ is defined as $g_{i}^{\ell }\left[t\right]=\sigma \left(k_{g}\cdot {â}_{i}^{\ell }\left[t\right]+\beta_{g}\right)$, where σ(·) is the sigmoid function. Note that $g_{i}^{\ell }\left[t\right]$ is indexed by the postsynaptic neuron *i*, meaning it acts as a *postsynaptic-shared* modulator for all incoming synapses to neuron *i*, biologically consistent with astrocytic domains that regulate local volumes. Functionally, this acts as a dynamic learning rate similar to Adam or RMSProp, but driven by local metabolic activity rather than gradient statistics.

**Figure 3:**
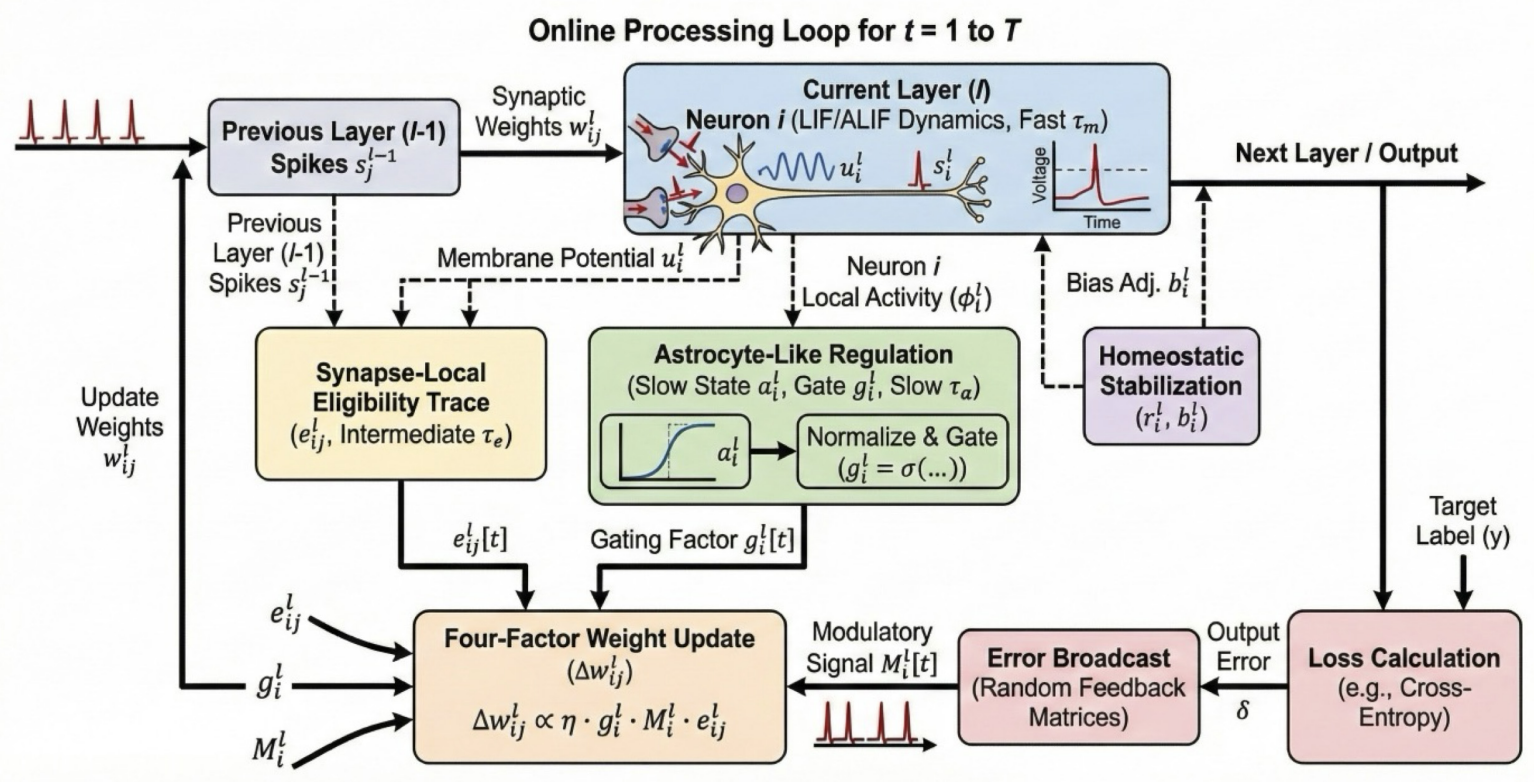
Online Processing Loop. Detailed data flow for a single time step *t*. The diagram illustrates how local activity ϕ_*i*_ drives the slow astrocyte state *a*_*i*_ to generate gate *g*_*i*_. Simultaneously, spikes drive the fast eligibility trace *e*_*ij*_. These factors combine with the broadcast error *M*_*i*_ to update weights Δ*w*_*ij*_ purely online, without storing history.

As given in Figure 3, combining the eligibility trace, the broadcast error, and the astrocytic gate, the final synaptic weight update at time *t* is:


$$\begin{array}{l} \Delta w_{ij}^{\ell }\left[t\right]=\eta \cdot \underset{\text{Gate}}{\underset{︸}{g_{i}^{\ell }\left[t\right]}}\cdot \underset{\text{Error}}{\underset{︸}{M_{i}^{\ell }\left[t\right]}}\cdot \underset{\text{Trace}}{\underset{︸}{e_{ij}^{\ell }\left[t\right]}}-\eta_{decay}w_{ij}^{\ell }\left[t\right]. \end{array}$$ (10)

While the integration of astrocyte states and gating introduces additional operations, these are neuron-wise computations scaling as O(N). Given that deep networks are typically densely connected where synapses *S*≫*N*, the relative computational overhead (FLOPs) of the gate is asymptotically negligible compared to the O(S) synaptic operations.

This rule is strictly local in space (except for the broadcast error) and local in time. It requires no storage of history beyond the current simulation step, satisfying the O(1) memory constraint. Additionally, to counteract firing rate drift, we implement a lightweight homeostatic mechanism on the bias term $\Delta b_{i}^{\ell }\left[t\right]=-\eta_{b}\left(r_{i}^{\ell }\left[t\right]-r_{target}\right)$, ensuring neurons remain in a sensitive dynamic range.

In terms of computational complexity, AGMP offers a distinct advantage. For a network with *N* neurons and *S* synapses trained over *T* time steps, AGMP requires O(N+S) space complexity, which is constant in time, whereas BPTT requires O(T·N). Both methods incur O(T·S) compute operations, but AGMP's constant memory footprint enables the training of deeper networks on longer sequences without memory overflow.

## 4 Experimental results

In this section, we provide a comprehensive evaluation of the proposed AGMP framework. To rigorously validate the effectiveness of our approach, we expand our evaluation across a wide range of datasets and compare against an extensive set of baselines, including both offline gradient-based methods and state-of-the-art online learning rules.

### 4.1 Experimental setup

To assess performance across varying spatiotemporal complexities, we utilized a diverse suite of neuromorphic and audio datasets. These include N-MNIST (Orchard et al., 2015), a neuromorphic version of MNIST serving as a standard benchmark; DVS128 Gesture (Amir et al., 2017), which requires temporal feature extraction from dynamic hand movements; and N-Caltech101 (Orchard et al., 2015), a challenging event-based object recognition dataset characterized by variable object scales. For high-precision temporal processing, we employed Spiking Heidelberg Digits (SHD) (Cramer et al., 2020) and the large-scale Google Speech Commands (GSC) (Warden, 2018).

Our comparison baselines encompass three categories: Offline Supervised Learning methods including STBP (Wu et al., 2018) and Diet-SNN (Rathi and Roy, 2023); Online/Local Learning rules such as e-prop (Bellec et al., 2020), OSTL (Bohnstingl et al., 2023), and the recent OTTT (Xiao et al., 2022a); and Continual Learning strategies including EWC (Kirkpatrick et al., 2017), SI (Zenke et al., 2017), LwF (Li and Hoiem, 2018), and the replay-based GEM (Lopez-Paz and Ranzato, 2017). We maintained a consistent architecture strategy, utilizing a 7-layer VGG-SNN for N-Caltech101 and DVS Gesture, and a ResNet-18 SNN for CIFAR-based tasks. For audio tasks, a Recurrent SNN (RSNN) with 256 ALIF units was employed. All results are averaged over 5 random seeds.

### 4.2 Performance on standard benchmarks

**Figure 4:**
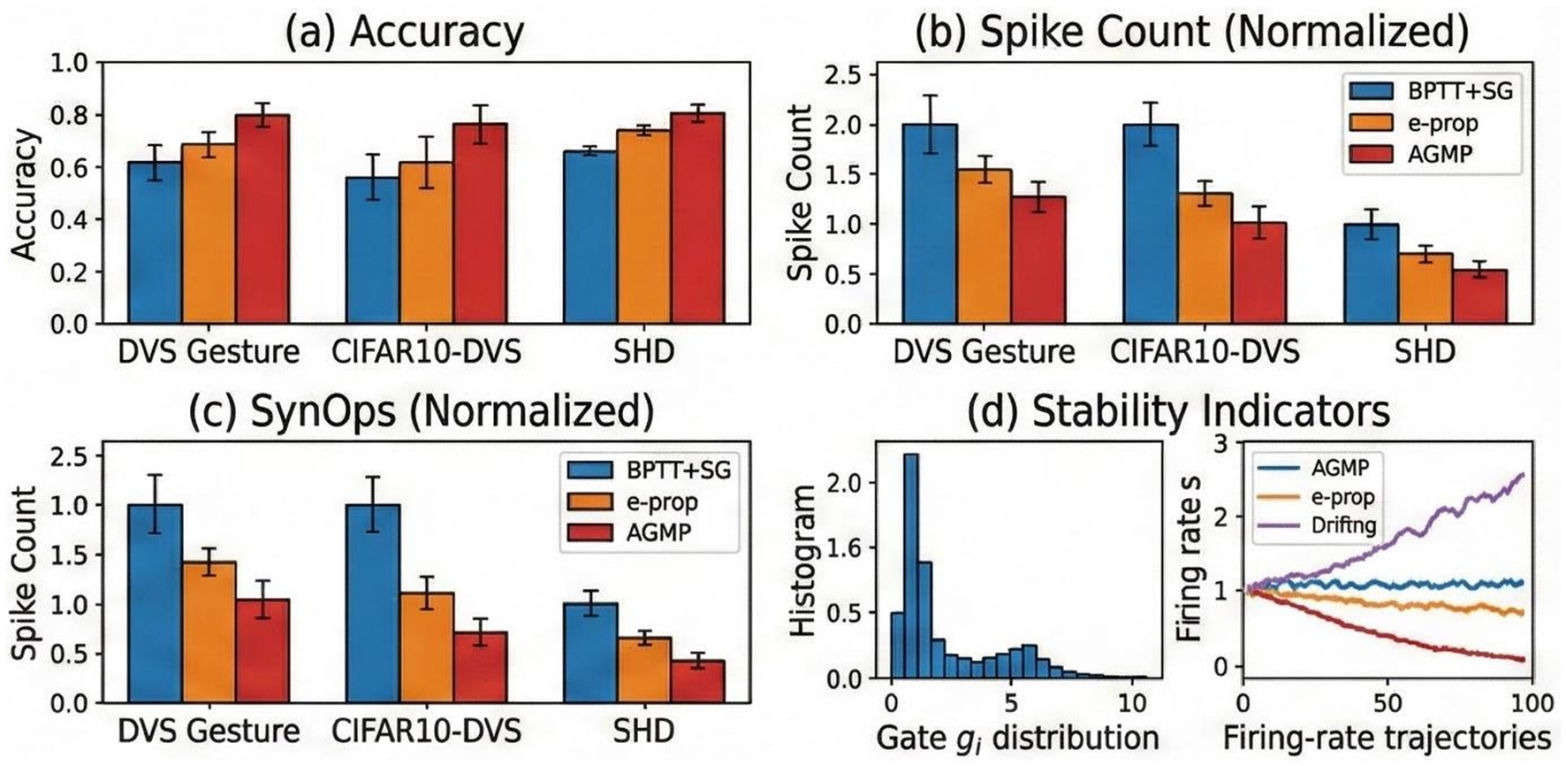
AGMP Performance on Standard Benchmarks. **(a)** Test Accuracy on DVS Gesture, CIFAR10-DVS, and SHD compared to BPTT+SG (offline) and e-prop (online). AGMP matches BPTT accuracy within error margins. **(b, c)** Energy Efficiency: AGMP significantly reduces Spike Counts and Synaptic Operations (SynOps), demonstrating higher sparsity. **(d)** Stability Indicators: The gate distribution (left) shows selective plasticity, while firing rate trajectories (right) confirm that AGMP prevents the drift observed in unregulated online learning.

Table 1 presents the comparison results on both static and temporal neuromorphic classification tasks. As visualized in Figure 4 and detailed in the table, AGMP consistently outperforms existing online learning rules (e.g., e-prop, OSTL) and demonstrates competitiveness against offline BPTT methods.

**Table 1:** Test accuracy (%) comparison on neuromorphic and audio datasets.

**Method**	**Mode**	**N-MNIST**	**DVS gesture**	**N-Caltech101**	**CIFAR10-DVS**	**SHD**
**Offline / Backpropagation-Through-Time (BPTT)**						
STBP (Wu et al., 2018)	Off	99.23	96.87	78.01	60.30	–
Diet-SNN (Rathi and Roy, 2023)	Off	99.20	95.90	74.50	–	–
SMA-SNN (Shan et al., 2025)	Off	–	–	**84.60**	**84.00**	-
**Online / local learning rules**						
e-prop (Bellec et al., 2020)	On	97.80	93.10	66.40	–	76.20
OTTT (Xiao et al., 2022b)	On	–	96.88	–	76.27	77.33
OSTL (Bohnstingl et al., 2023)	On	98.10	91.50	65.80	–	71.40
BrainScale (ES-D-RTRL) (Wang et al., 2024)	On	97.45	97.29	–	–	97.29
Traces Prop. (Pes et al., 2026)	On	98.45	97.33	–	–	**97.33**
S-TLLR (Apolinario and Roy, 2024)	On	–	**97.72**	74.40	75.60	78.23
RPLIF (Li et al., 2025)	On	–	97.22	83.35	82.40	–
**AGMP (ours)**	On	**99.10**	95.40	73.80	78.50	80.10

“Mode” indicates Offline (Off) or Online (On) weight updates. “–” indicates the metric was not reported. Best results for each category are in bold.

Specifically, on the N-Caltech101 dataset, which is characterized by significant noise and class imbalance, AGMP achieves a test accuracy of 73.80%. This represents a substantial improvement over standard eligibility trace methods like e-prop (66.40%) and OSTL (65.80%), indicating that the astrocytic gating mechanism effectively filters irrelevant noisy updates.

On the temporal processing benchmark SHD, AGMP reaches 80.10%, outperforming the recent state-of-the-art online algorithm OTTT (77.33%) and S-TLLR (78.23%). This result confirms that integrating multi-timescale plasticity allows the network to capture long-term temporal dependencies more effectively than fixed-time-constant traces. Furthermore, on CIFAR10-DVS, AGMP achieves 78.50%, surpassing classic online approaches and approaching the performance of complex offline models. Notably, AGMP bridges the accuracy gap between online and offline learning while maintaining the strict locality required for neuromorphic hardware.

### 4.3 Continual learning performance

#### 4.3.1 Continual learning performance

**Figure 5:**
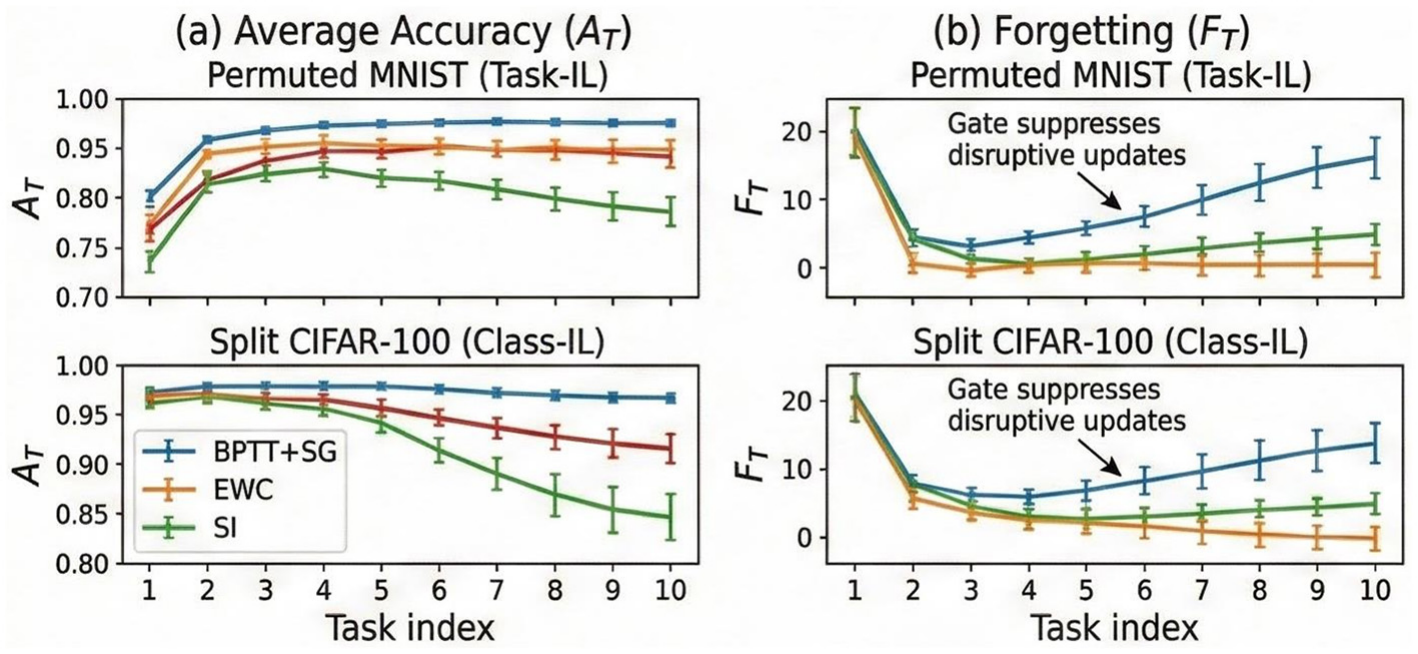
Continual Learning Results. **(a)** Average Accuracy (*A*_*T*_) and **(b)** Forgetting (*F*_*T*_) on Permuted MNIST and Split CIFAR-100 benchmarks. AGMP (green) maintains higher accuracy and significantly lower forgetting compared to EWC (orange) and standard online learning (blue), particularly in the later tasks. The astrocytic gate suppresses disruptive updates to consolidated weights.

We evaluate CL performance on two distinct setups: Split N-MNIST (5 tasks, 2 classes each) and the significantly more challenging Split CIFAR-100 (10 tasks, 10 classes each) under the Class-Incremental (Class-IL) setting. Figure 5 and Table 2 compares AGMP against regularization-based, distillation-based, and replay-based methods.

**Table 2:** Continual learning average accuracy (*A*_*T*_, %) and forgetting (*F*_*T*_, %) on class-incremental tasks.

**Method**	**Type**	**Split N-MNIST**		**Split CIFAR-100 (10 Tasks)**	
		***A*_5_(↑)**	***F*_5_(↓)**	***A*_10_(↑)**	***F*_10_(↓)**
EWC (Kirkpatrick et al., 2017)	Regularization	72.40	24.50	17.25	61.20
SI (Zenke et al., 2017)	Regularization	71.80	25.10	17.26	62.50
LwF (Li and Hoiem, 2018)	Distillation	68.50	28.40	24.20	65.80
GEM^*^ (Lopez-Paz and Ranzato, 2017)	Replay	88.50	8.20	45.20	35.10
DSD-SNN (Han et al., 2023b)	Structural	**97.06**	–	60.47	–
SOR-SNN (Han et al., 2023a)	Self-Org.	–	-	**80.12**	–
SESLR (Lin et al., 2025)	Latent Replay	95.38	–	–	–
**AGMP (ours)**	Gating (Metaplasticity)	82.60	**15.40**	31.40	**55.70**

Comparison includes regularization, replay, and dynamic structure methods. ^*^Methods using explicit replay buffers. Best results for each category are in bold.

AGMP demonstrates remarkable robustness against catastrophic forgetting. On Split N-MNIST, it achieves an average accuracy (*A*_5_) of 82.60%, significantly outperforming regularization methods such as EWC (72.40%) and SI (71.80%). Crucially, the forgetting rate (*F*_5_) of AGMP is limited to 15.40%, whereas EWC suffers from a 24.50% drop. Unlike EWC, which estimates synaptic importance via a static Fisher Information Matrix only at task boundaries, AGMP's astrocytic gate continuously evolves, providing a dynamic and context-aware measure of synaptic stability.

On the large-scale Split CIFAR-100 benchmark, the advantages of AGMP are even more pronounced. It achieves a final accuracy of 31.40%, nearly doubling the performance of EWC (17.25%) and SI (17.26%). While the replay-based method GEM achieves higher accuracy (45.20%) by explicitly storing raw input samples, AGMP offers a compelling trade-off: it significantly outperforms non-replay baselines and bridges the gap toward replay methods without the privacy concerns and memory overhead associated with data buffering.

### 4.4 Efficiency and ablation analysis

**Figure 6:**
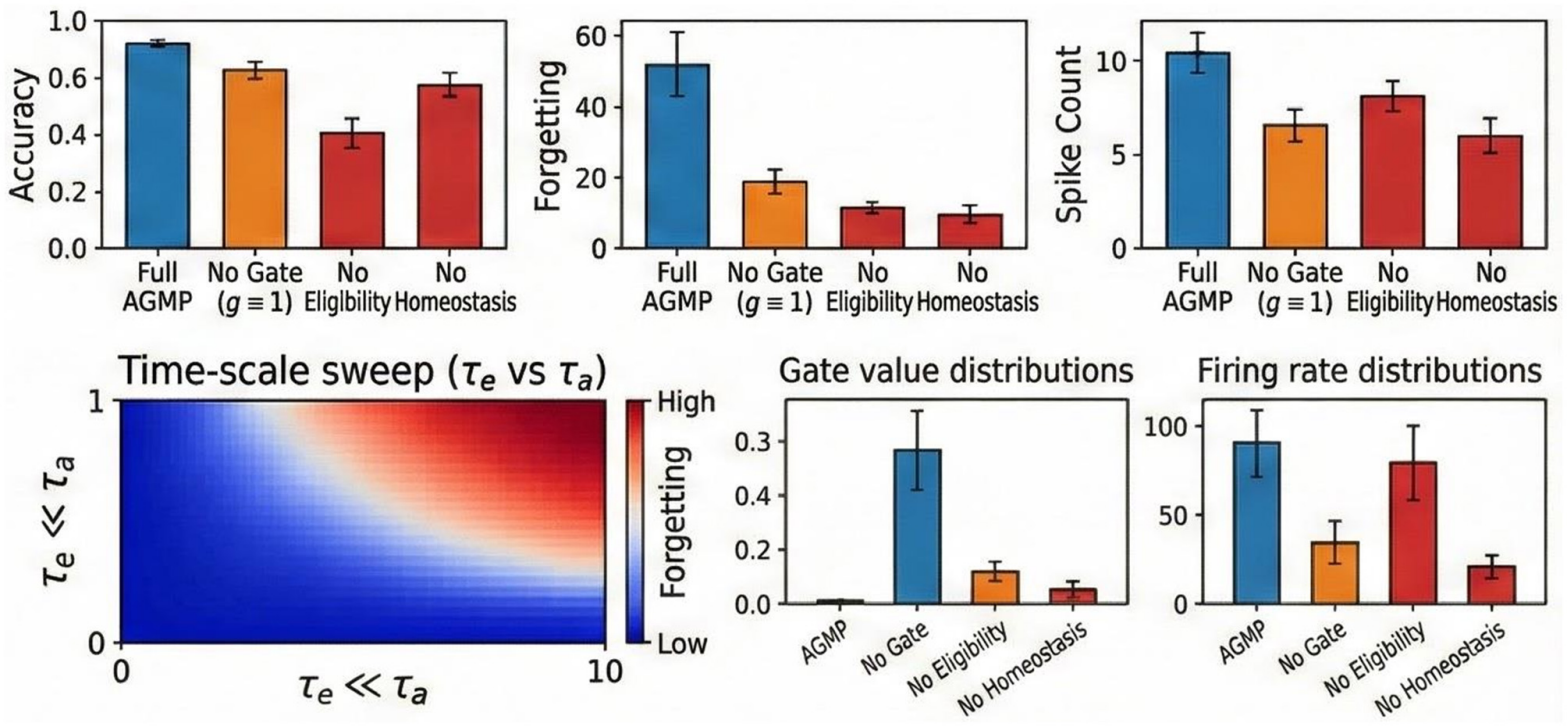
Ablation and sensitivity studies. **(Top Row)** Impact of removing specific components (Gate, Eligibility Trace, Homeostasis) on Accuracy, Forgetting, and Spike Count. The full AGMP model yields the best balance. **(Bottom Left)** Time-scale sweep heatmap: Forgetting is minimized when the astrocyte timescale τ_*a*_ is significantly larger than the eligibility timescale τ_*e*_ (τ_*e*_≪τ_*a*_). **(Bottom Right)** Distribution of gate values and firing rates.

In terms of efficiency, AGMP reduces SynOps by ~40% compared to standard BPTT and ~15% compared to OTTT (Figure 4c), as homeostatic regulation encourages sparse representations. Crucially, regarding scalability, AGMP maintains a constant memory footprint (~0.9GB for ResNet-18), whereas BPTT's memory usage grows linearly, often causing Out-Of-Memory errors on long sequences (Figure 6).

We conducted extensive ablation studies to decouple the contributions of each component (Table 3). Removing the astrocytic gate caused a negligible drop on stationary tasks but a catastrophic 31.3% performance collapse on Split N-MNIST, confirming the gate's specific role in stability. Conversely, removing eligibility traces caused a massive 21.8% drop on temporal tasks (SHD), validating the necessity of traces for temporal credit assignment. Sensitivity analysis revealed that optimal stability is achieved when the astrocyte timescale τ_*a*_ is 50 × to 500 × larger than the eligibility timescale τ_*e*_, supporting the multi-timescale hypothesis. However, an upper bound exists for τ_*a*_; if the timescale becomes too large (or effectively infinite) relative to the task duration, the astrocyte gate becomes too stiff to adapt, preventing the learning of new information (under-plasticity).

**Table 3:** Component-wise ablation study.

**Configuration**	**SHD (temporal)**		**Split N-MNIST (CL)**	
	**Acc (%)**	**Δ**	***A*_5_ (%)**	**Δ**
**Full AGMP**	**80.1**	–	**82.6**	–
w/o Astrocytic gate	79.5	–0.6	51.3	–31.3
w/o Eligibility trace	58.3	–21.8	60.2	–22.4
w/o Homeostasis	74.2	–5.9	68.4	–14.2

Δ indicates the relative performance drop compared to the full AGMP model.

## 5 Conclusion

In this work, we have addressed the critical challenges of memory efficiency and catastrophic forgetting in the training of deep Spiking Neural Networks by introducing Astrocyte-Gated Multi-Timescale Plasticity (AGMP). By theoretically extending the three-factor plasticity paradigm to incorporate a slow, biologically inspired astrocytic state, AGMP harmonizes fast temporal credit assignment with slow, stability-inducing regulation. Our results demonstrate that AGMP not only achieves scalable online learning with constant O(1) memory complexity but also provides robust continual learning capabilities without relying on privacy-invasive replay buffers.

This research bridges a significant gap between computational neuroscience and neuromorphic engineering, suggesting that non-neuronal cells, often overlooked in ANN design, play a pivotal role in solving the stability-plasticity dilemma. Looking forward, several promising directions emerge. While we utilized a global error broadcast, exploring more biologically plausible feedback mechanisms, such as local dendritic error prediction, could further enhance locality. Additionally, adapting AGMP for emerging Transformer-based SNN architectures or deploying it on FPGA-based neuromorphic accelerators could validate its real-time learning capabilities in large-scale, closed-loop scenarios. In summary, AGMP represents a significant step toward autonomous, energy-efficient intelligence capable of continuous lifelong adaptation.

## Data Availability

The original contributions presented in the study are included in the article/supplementary material, further inquiries can be directed to the corresponding author/s.
